# HIV-1 Subtype C Phylodynamics in the Global Epidemic

**DOI:** 10.3390/v2010033

**Published:** 2010-01-07

**Authors:** Vlad Novitsky, Rui Wang, Stephen Lagakos, Max Essex

**Affiliations:** 1 Department of Immunology and Infectious Diseases, Harvard School of Public Health AIDS Initiative, Harvard School of Public Health, Boston, MA, USA; E-Mail: messex@hsph.harvard.edu (M.E.); 2 Botswana–Harvard AIDS Institute, Gaborone, Botswana; 3 Department of Biostatistics, Harvard School of Public Health, Boston, MA, USA; E-Mails: rwang8@partners.org (R.W.); lagakos@sdac.harvard.edu (S.L.)

**Keywords:** HIV-1 subtype C, consensus sequence, amino acid frequency, Gag, *gag* phylogeny, CTL epitopes, time of MRCA

## Abstract

The diversity of HIV-1 and its propensity to generate escape mutants present fundamental challenges to control efforts, including HIV vaccine design. Intra-host diversification of HIV is determined by immune responses elicited by an HIV-infected individual over the course of the infection. Complex and dynamic patterns of transmission of HIV lead to an even more complex population viral diversity over time, thus presenting enormous challenges to vaccine development. To address inter-patient viral evolution over time, a set of 653 unique HIV-1 subtype C *gag* sequences were retrieved from the LANL HIV Database, grouped by sampling year as <2000, 2000, 2001–2002, 2003, and 2004–2006, and analyzed for the site-specific frequency of translated amino acid residues. Phylogenetic analysis revealed that a total of 289 out of 653 (44.3%) analyzed sequences were found within 16 clusters defined by aLRT of more than 0.90. Median (IQR) inter-sample diversity of analyzed *gag* sequences was 8.7% (7.7%; 9.8%). Despite the heterogeneous origins of analyzed sequences, the gamut and frequency of amino acid residues in wild-type Gag were remarkably stable over the last decade of the HIV-1 subtype C epidemic. The vast majority of amino acid residues demonstrated minor frequency fluctuation over time, consistent with the conservative nature of the HIV-1 Gag protein. Only 4.0% (20 out of 500; HXB2 numbering) amino acid residues across Gag displayed both statistically significant (p<0.05 by both a trend test and heterogeneity test) changes in amino acid frequency over time as well as a range of at least 10% in the frequency of the major amino acid. A total of 59.2% of amino acid residues with changing frequency of 10%+ were found within previously identified CTL epitopes. The time of the most recent common ancestor of the HIV-1 subtype C was dated to around 1950 (95% HPD from 1928 to 1962). This study provides evidence for the overall stability of HIV-1 subtype C Gag among viruses circulating in the epidemic over the last decade. However selected sites across HIV-1C Gag with changing amino acid frequency are likely to be under selection pressure at the population level.

## 1. Introduction

The propensity of HIV to generate mutations and escape from immune pressure leads to considerable intra-host viral diversification over time. Upon transmission to a new host, the virus may restore its wild-type status through reverse mutations, and acquire new escape mutations in response to immune pressure from the new host. These dynamic processes underline the perpetual virus–host interactions over the endless chain of virus transmissions. Increasing HIV diversity impacts most biomedical prevention and therapeutic strategies, and especially affects the design of HIV vaccine antigens.

Because of the complex nature of transmission of HIV in a population, an understanding of the dynamics of HIV evolution at the population level is particularly challenging. To shed additional light on this, we assess the diversity and time changes of the HIV-1 structural protein Gag, as this is one of the most attractive targets for HIV vaccine design. Recent studies have demonstrated that HIV-1 Gag can induce potent virus-specific T cell responses, and provided evidence that the breadth, magnitude, and functional profile of such immune responses are associated with control of viral replication, low viral set point, and better disease prognosis [1–15]. Therefore, better understanding of viral dynamics and the *in vivo* mutational pathways in Gag could further the rational design of an HIV-1 vaccine. We also focus on HIV-1 subtype C because it is the predominant viral subtype in the worldwide HIV/AIDS epidemic, and the HIV subtype associated with the highest incidence and prevalence rates during heterosexual transmission.

Knowledge of viral mutations directly selected due to their fitness cost and immune pressure could aid in the discovery of new approaches to efficient control of HIV. Recent studies addressing the natural course of HIV infection and/or utilizing SIV models have shown that virus-specific CD8+ T cell responses and neutralizing antibodies are main determinants of HIV evolution on the population level in the HIV/AIDS epidemic [16–21].

Phylodynamic studies may help to reveal the biological mechanisms underlying observed HIV evolution and thereby enable us to better control the HIV/AIDS epidemic. Analysis of viral mutational pathways on a population level could lead to better understanding of the limits of viral sequence variation and to identification of viral evolutionary space in the HIV/AIDS epidemic. The goal of the current analyses was to investigate how HIV-1 subtype C variation in *gag*/Gag is modulated by epidemic dynamics in order to better understand how stable subtype C consensus is over time, and whether amino acid toggling [21] and/or stochastic mutations [22] affect its structure and dynamics.

## 2. Results and Discussion

The study addressed the following four aspects of viral diversity: (1) phylogeny and diversity of HIV-1 subtype C *gag* over time; (2) extent of amino acid frequency change in Gag over time at specific sites; (3) relationships between changing amino acids in HIV-1 subtype C Gag and CTL epitopes; and (4) the date of HIV-1 subtype C divergence.

### 2.1. The *in vivo* dynamics of gag diversity

The phylogeny of HIV-1 subtype C as the dominant virus in the HIV/AIDS epidemic has been the subject of considerable recent interest. In this study we address the following questions: (*i*) Are there lineages or sub-clades within HIV-1 subtype C *gag* sequences? If so, are sub-clusters within HIV-1 subtype C associated with geographic origin or time of sampling? Is there a segregation of the C and C’ sub-clades from Ethiopia? Are there distinguishable lineages of viruses originating from Southern Africa? (*ii*) What is the match between the traditional tree-based phylogenetic analysis and the split network analysis? If there is a conflict, is there evidence for recombination events? (*iii*) What is the extent of viral diversity of contemporaneous HIV-1 subtype C sequences? What is the dynamic of viral diversity within *gag* over time?

**Figure 1.**
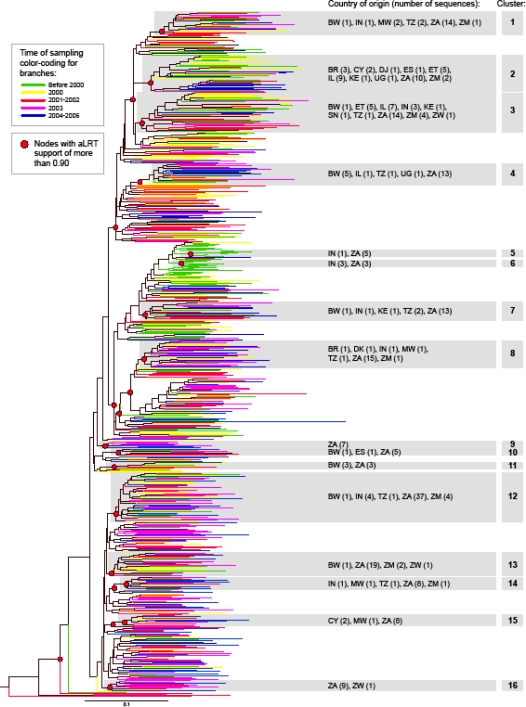
Phylogenetic relationships between HIV-1 subtype C *gag* sequences. Phylogenetic tree was constructed by PhyML. Color branches represent year of sampling by group. Nodes with significant aLRT support are highlighted.

To analyze phylogenetic relationships between HIV-1 subtype C *gag* sequences, we determined the maximum likelihood tree by PhyML [23], with the approximate Likelihood Ratio Test as a statistical test for branch support (using a criterion of aLRT>0.90). The maximum likelihood tree is presented in Figure 1 with color-coded branches corresponding to different times of sampling, and highlighted nodes with aLRT support of more than 0.90. A total of 16 terminal clusters with more than five sequences were found with an aLRT value of more than 0.90 (Figure 1). The median (IQR) number of sequences in clusters was 15 (7; 22), and ranged from 6 (clusters 5, 6, and 11) to 47 (cluster 12). A total of 289 out of 653 (44.3%) analyzed sequences were found in clusters within HIV-1 subtype C, which was consistent with our previous report on intra-subtype lineages in subtype C viruses [24].

HIV-1 subtype C *gag* sequences representing different time points of sampling were scattered throughout the phylogenetic tree without any suggestion of branching topology being related to the time of sampling. Although two small clusters (5 and 6) were comprised exclusively of Indian and South Africa sequences sampled in 1999, earlier time points were relatively rare at the bottom half of the tree. Cluster 16 included only sequences sampled in 2001 and later. Although clusters 5, 6, and 16 include small numbers of sequences, they might suggest ongoing extinction and evolution of virus intra-subtype lineages in the epidemic, highlighting the importance of sequence monitoring.

Analysis of sequence distribution by the country of origin reveals that viruses from South Africa were present in 14 of the 16 clusters, suggesting that extensive sequence analysis is important, and is able to reveal distinct lineages within HIV-1 subtype C [24]. Ten of 12 available sequences from Ethiopia were equally split between clusters 2 and 3, supporting previous findings of C and C’ viruses in this country [25–30]. Interestingly, 16 of 22 sequences from Israel were also split between clusters 2 (nine sequences) and 3 (seven sequences) together with the Ethiopian sequences, further supporting the existence of distinct lineages within HIV-1 subtype C. Indian sequences did not form a single cluster but were present in multiple clusters, suggesting ongoing diversification.

**Figure 2.**
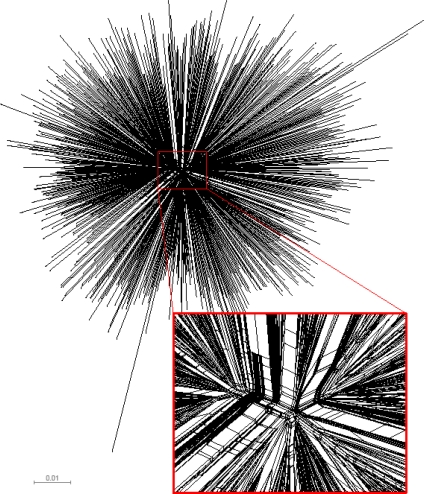
Phylogenetic network of HIV-1 subtype C *gag* sequences (n=653). The presented split network was generated by SplitsTree v4 [32,33] using the NeighborNet approach. To highlight multiple splits and parallel branching, the central part of the split network is enlarged.

We examined the congruence between the topologies of unrooted single phylogenetic tree and split network to identify potential deviations from the tree-like structure of analyzed *gag* sequences. Computation of split networks was performed using the NeighborNet approach which represents a “hybrid” between the neighbor-joining and split decomposition methods [31]. The resulting phylogenetic network produced by SplitsTree v4 [32,33] is presented in Figure 2, and depicts a star-like phylogeny of analyzed HIV-1 subtype C *gag* sequences. The central part of the phylogenetic network highlights multiple splits and parallel branching, which is consistent with the recent report of HIV-1 envelope sequences from subtype B collected in the USA [20]. The observed pattern is a signature for a potential conflict between phylogenetic tree and split network. The presence of splits provides evidence that the analyzed set of HIV-1 subtype C *gag* sequences possesses some amount of phylogenetic signal in the data that cannot be explained adequately by a single tree. However, since the splits are present predominantly in the central part of the split network and the extent of splits is moderate, the tree-based phylogenetic analysis is valid. It is likely that the recombination events can be found in the analyzed set of *gag* sequences, and if so, recombination could explain the presence of splits. Further analysis using alternative phylogenetic networks including recombination networks should help to reconstruct a detailed evolutionary history of HIV-1 subtype C sequences.

**Figure 3.**
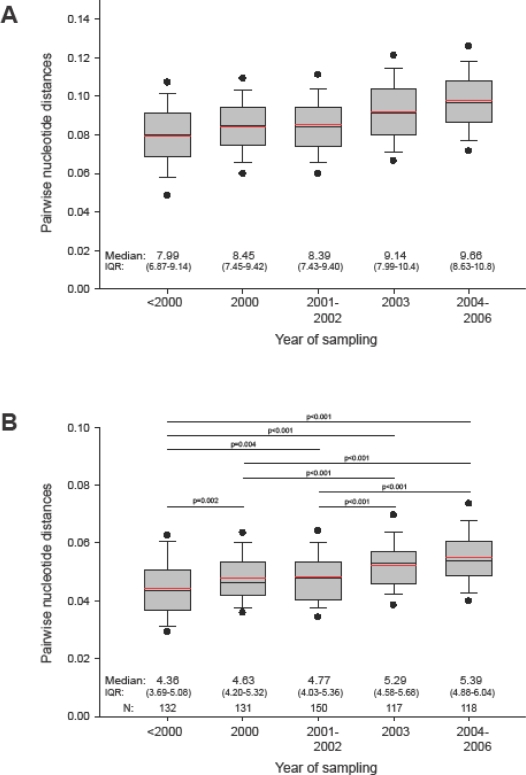
Pairwise distances of HIV-1 subtype C *gag* sequences collected at different time points. In the box plots: The boundary of the box closest to zero indicates the 25^th^ percentile, a black line within the box marks the median value, a red line within the box shows the mean, and the boundary of the box farthest from zero indicates the 75^th^ percentile. Whiskers above and below the box indicate the 10^th^ and 90^th^ percentiles. Points above and below the whiskers indicate the 5^th^ and 95^th^ percentiles. Five groups in each graph correspond to the time of sampling. **A:** Pairwise distances within group by sampling time. **B:** Pairwise distances to HIV-1 subtype C consensus. Comparisons between groups are based on Mann-Whitney sum rank test.

To assess viral diversity in HIV-1 subtype C epidemic, we analyzed *gag* pairwise distances within the entire set of sequences and within subsets of sequences grouped by sampling year. The median (IQR) pairwise diversity of 653 analyzed subtype C *gag* sequences was 8.73% (7.65%; 9.84%), ranging from 0.07% to 23.9%. The mean value of the overall diversity in *gag* was 8.8%. Figure 3A displays the diversity within subsets of sequences grouped by sampling year. Comparison of pairwise distances over different sampling periods reveals a statistically significant (p<0.001 by Mann-Whitney Rank Sum test) increase of viral diversity in HIV-1 subtype C *gag* sequences in the epidemic of about 1.5% over the last decade (slope of 0.00185 per year).

To evaluate relationships between HIV-1 subtype C consensus (derived from LANL) and actual sequences, we analyzed pairwise distances between subtype C *gag* consensus and analyzed sequences. As expected, median (IQR) pairwise distance to subtype consensus was about half of the median value among all sequences, 4.88% (4.22%; 5.57%), and had a smaller range, from 2.05% to 12.03%. Similarly, distances between subtype consensus and subsets of sequences sampled at different time points were approximately half the corresponding distances among subsets of sequences (Figure 3B). There was a gradual increase over the decade (slope of 0.00124 per year), with statistically significant differences among the more remote subsets, providing evidence for overall diversification of the circulating viral sequences in the HIV-1 subtype C epidemic from the previously established subtype consensus sequence. The data also suggests the need for a regular update of subtype consensus so as to be able to adequately represent circulating viruses. Although our analysis revealed a star-like phylogeny of HIV-1 subtype C *gag* sequences sampled over decade of epidemic, the observed gradual increase in mean and median pairwise distances necessitates further screening to exclude virus evolving in any specific direction. A designed sample collection can also improve balanced representation of different geographic areas and minimize sampling bias in generating the consensus sequence.

The *F_ST_* statistics was used to assess divergence between earliest, before 2000, and the latest, 2004–2006, samples. Values of *F_ST_* were estimated by methods described by Hudson, Slatkin and Madison [34], Slatkin [35] and Hudson, Boos and Kaplan [36] and implemented by the HyPhy package [37]. The estimated *F_ST_* values were 0.027, 0.014, and 0.014 by three mentioned methods, respectively, suggesting lack of divergence between the earliest and the latest sample sets.

### 2.2. Amino acid frequency in HIV-1 subtype C Gag

To evaluate viral dynamics on a population level, we compared amino acid frequency in the HIV-1 subtype C Gag extended consensus sequences corresponding to different sampling times, before 2000, 2000, 2001–2002, 2003, and 2004–2006. Numbering of amino acid sites across Gag from 1 to 500 corresponded to the HXB2 numbering system (http://www.hiv.lanl.gov/). Three statistical approaches were used for analysis of amino acid frequencies at each residue, the chi-square test, the Cochran-Armitage trend test, and analysis of the ranges in amino acid frequency over time.

In the first analysis, we tested, for each residue position, whether the distribution of amino acid frequency changes over the five time intervals using the chi-square test for Rx5 tables, where R is the number of amino acid types. A total of 44 of 500 (8.8%) amino acid residues across Gag were identified with significant (p<0.05) changes in their frequency over time. Changing amino acid residues identified by the chi-square test were spread unevenly across Gag: 14 (32%) were located in Gag p17, 9 (21%) in p24, 2 (5%) in p2, 2 (5%) in p7, 10 (23%) in p1, and 7 (16%) in p6, accounting for 21 (48%) changing amino acid residues in p15.

In the second analysis, we tested, for each position, whether the frequency of the major amino acid changed monotonically (increasing or decreasing) over time, using the Cochran-Armitage trend test. With this analysis, a significant (p<0.05) trend was found in 75 (15%) of the 500 positions. The distribution of changing amino acid residues across Gag cleavage products was more even: 21 (28%) were found in Gag p17, 28 (37%) in p24, 2 (3%) in p2, 3 (4%) in p7, 12 (16%) in p1, and 9 (12%) in p6.

Recognizing that relatively small changes in amino acid frequency over time can sometimes yield statistically significant test results, the third analysis examined whether, for each position, the frequencies of the major amino acid at different time intervals differed by more than a specified amount. A total of 8 of 500 (1.6%) amino acid positions demonstrated a range of frequencies over time that is more than 20%, with maximum range of 36% at amino acid position 11 in p17 between gradually reducing Gly and increasing Glu on a background of low frequency of Thr, Lys, Asp, and Ala. In addition, a total of 41 (8.3%) amino acid positions exhibited ranges in frequency between 10% and 20%, while 98 (19.9%) amino acid positions exhibited ranges between 5% to 10%. Analysis of amino acid toggling on a population level in the context of dominant HLA alleles can be an important direction to be pursued in future studies.

**Figure 4.**
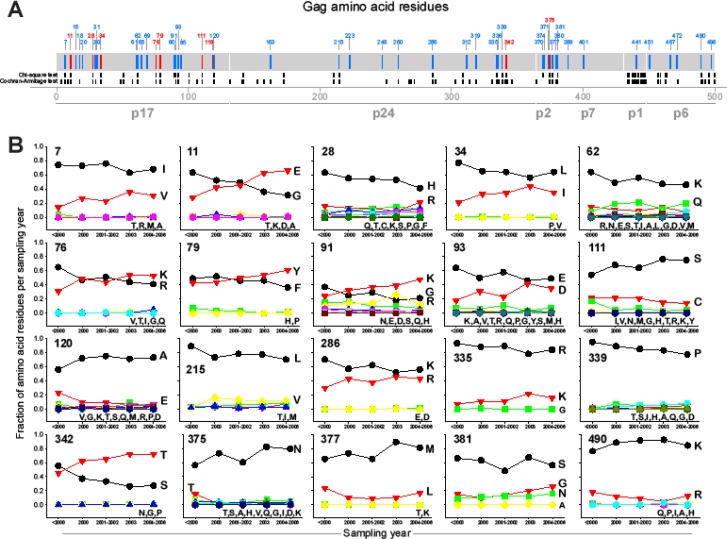
Changing amino acid residues in HIV-1 subtype C Gag. **A:** Overview of changing amino acid residues across Gag cleavage products. Location of changing amino acids is shown in relation to HXB2 numbering. Changing amino acid sites with frequency change of more than 20% are shown in red, and sites with frequency change between 10% and 20% are shown in blue. Location of changing amino acids detected by chi-square test and Cochran-Armitage trend test is depicted in black under the Gag bar. **B:** Dynamics of amino acid frequency at top 20 sites (significant changes by all three methods). Number in the upper left corner in each graph depicts location in relation to HXB2 numbering.

Combining the results of the statistical tests of heterogeneity/trend and the analyses of magnitude of change in amino acid frequency resulted in only 20 of 500 (4.0%) positions which demonstrated statistically significant changes by the heterogeneity and trend test as well as a range of amino acid frequencies of at least 10%. Figure 4A highlights amino acid positions across Gag that showed significant changes in amino acid frequency by any method. Figure 4B provides details of amino acid frequency dynamics at 20 positions across Gag that showed significant changes by all three methods.

To distinguish trends of specific amino acid increase (decrease), which may be more likely as a result of directed selection on a population level, from fluctuations in amino acid frequency, which is more likely to be a sampling issue, we analyzed the slopes of increasing and decreasing amino acids within a subset of 20 amino acid positions with significant changes over time. The median (IQR) increasing amino acid slope within the subset was 0.017 (0.012; 0.023), ranging from 0.003 to 0.042. This translates into an average increasing rate of selected amino acids within HIV-1 subtype C of 17% (12%; 23%) over the decade. The median (IQR) decreasing amino acid slope within the subset of 20 amino acid positions with significant changes over time was −0.017 (−0.020; −0.014), ranging from −0.041 to −0.008, which corresponds to an average 10-year decline 17% (14%, 20%) of selected amino acids.

Taken together, these results indicate a remarkable stability of HIV-1 subtype C consensus over the last decade in the HIV-1 subtype C epidemic. A majority of sites across HIV-1 subtype C Gag showed minimal changes over time and frequency fluctuations within small ranges. A relatively small number of sites across HIV-1 subtype C Gag, 20 (4.0%), demonstrated statistically significant changes in amino acid frequency over time that were confirmed by three applied methods (chi-square test, Cochran-Armitage test, and analysis of ranges). Patterns of changing amino acids within this subset were consistent with expected positive or negative slopes.

### 2.3. Synonymous and non-synonymous changes

**Figure 5.**
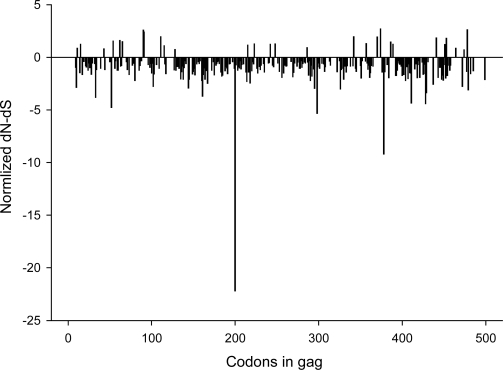
Normalized dN-dS values across gag codons. P-values were derived from a two-tailed extended binomial distribution. A total of 31 positively and 242 negatively selected sites were found.

To identify sites under positive or negative selection in HIV-1 subtype C *gag* we estimated the rates of non-synonymous and synonymous changes at each site by using single-likelihood ancestor counting method (SLAC) as described by Kosakovsky Pond and Frost [38]. This analysis revealed 31 positively selected sites and 242 negatively selected sites that were supported by p-valued of less than 0.05. All codon positions with significant (p<0.05) positive or negative selection are outlined in Figure 5. Seven of the sites presented in Figure 4B were found to be under positive (diversifying) selection in the SLAC analysis (positions 11, 62, 91, 111, 215, 286, and 342). Two of the sites presented in Figure 4B, positions 28 and 34, were under negative (purifying) selection by SLAC.

### 2.4. Changing amino acids and CTL epitopes

It is believed that changes in amino acid frequency in the global HIV-1 subtype C consensus over time are driven by cumulative immune pressure on the population level. MHC class I HLA alleles that are associated with control of HIV infection impose the strongest pressure at viral epitopes [20,39]. Therefore, it is plausible that changing amino acid residues should be located within the virus-specific CTL epitopes. To test this assumption, the Los Alamos National Laboratory HIV immunology database (http://www.hiv.lanl.gov/content/immunology; accessed on 14 August 2009) was screened for known human CTL epitopes identified in the context of HIV-1 subtype C infection irrespective of their MHC class I HLA alleles restriction, and the location of changing amino acids was matched with the retrieved epitopes.

A relatively high fraction of amino acid residues with changing frequency of 10% and higher, 29 of 49 (59.2%), was found within previously identified CTL epitopes, including five of eight amino acid residues with changing frequency of 20% and higher. Relationships between amino acids with frequency change of ≥10% and involved CTL epitopes are presented in Tables 1 (frequency change of ≥20%) and 2 (frequency change of 10–20%). The location of amino acids is shown within the CTL epitopes, and epitopes with multiple changing amino acids are highlighted. Amino acids with changing frequency but without matching CTL epitopes reflect gaps in our knowledge regarding HIV-1 Gag-specific T cell responses. Further studies are warranted to identify mechanisms that drive changes of amino acid frequency at the population level.

The status of a specific well-studied CTL epitopes in HIV-1 Gag is of particular interest. Specifically, analysis was performed for 30 well defined epitopes in Gag with HLA-associated polymorphic residues analyzed recently by Brumme *et al* [40]. A subset of 15 of 30 (50%) epitopes included amino acids with changing frequency of 10% and higher, although some changing amino acids differed from the HLA-associated polymorphisms within epitopes. For example, within the epitope SL9, SL**Y**NTV**A**TL, Tyr at the third position has been a gradually increasing amino acid in HIV-1 subtype C over a decade (0.43 → 0.44 → 0.50 → 0.54 → 0.61), while Ala at the seventh position, which is considered to be an HLA-A02-associated residue, stayed relatively stable (0.95 → 0.98 → 0.97 → 1.0 → 0.97) over time. Although our analysis did not involve HLA typing data, the study results on viral mutations within optimized CTL epitopes are in line with finding by the Philip Goulder group on HLA-driven viral evolution [41].

### 2.5. Dating of HIV-1 subtype C divergence

**Figure 6.**
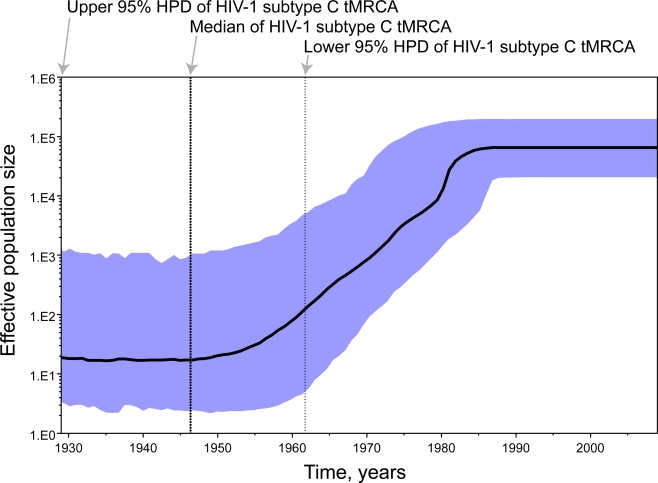
Bayesian skyline plot of HIV-1 subtype C. The upper 95% HPD, median, and lower 95% HPD of HIV-1 subtype C are projected on the time line. The bold black line traces the inferred median effective population size over time with the 95% HPD shaded in blue.

To assess the date of the HIV-1 subtype C divergence we estimated the time to the most recent common ancestor (tMRCA) of subtype C viruses in a subset of 138 *gag* sequences selected by country representation (limit was set to three sequences per country per year of sampling). A relaxed clock Bayesian MCMC coalescent framework analysis was implemented in BEAST v1.4.8 [42]. This approach incorporates phylogenetic uncertainty and accounts for the possibility of variable substitution rates among lineages and differences in the demographic history of the virus, sampling phylogenies and parameter estimates in proportion to their posterior probability [43–46]. Substitution rates were calibrated with analyzed *gag* sequences with known year of sampling. The median (95% highest posterior density interval, HPD, a Bayesian analog to a confidence interval) substitution rate in *gag* was estimated as 2.65×10^–3^ (1.87x10^–3^–3.49 ×10^–3^) substitutions per site per year. The different demographic/coalescent models gave similar estimates for HIV-1 subtype C tMRCA. The tMRCA (95% HPD) of HIV-1 subtype C was estimated at 1950 (1930–1962) based on a constant population size model and at 1948 (1928–1962) using the Bayesian skyline plot [47,48] (Figure 6), which is consistent with the estimated date of tMRCA of HIV-1 group M [44].

HIV-1 viruses evolved from a common ancestor circulating at the beginning of the twentieth century [44] that was acquired by humans through cross-species transmissions followed by a split into HIV-1 M group subtypes as a result of founder events [49,50]. We estimated the date of HIV-1 subtype C diversification around 1950 (1928–1962). These results are consistent with the notion that HIV-1 subtypes underwent several decades of independent evolution in humans [44,50–52] before reaching a sizable HIV/AIDS epidemic.

A deeper understanding of reasons for the flattening the HIV-1 subtype C population size after 1980 is of great importance. Theoretical considerations suggest that some sort of evolutionary constraint may exist for virus adaptation as a consequence of environmental, selective, genetic, or functional trade-offs (e.g., negative epistasis and pleotropy) that can limit viral evolution [53], and can be one of the potential causes for the flattering of the population size. However, this analysis could be confounded by the increasing frequency of intra-subtype recombinants that accompany epidemic growth.

## 3. Experimental Section

*Included HIV-1 subtype C gag sequences.* The extended HIV-1 subtype C consensus was generated as described elsewhere [24]. A total of 653 unique (one sequence per patient) HIV-1 subtype C sequences spanning more than 1,000 bp of *gag* were retrieved from the Los Alamos HIV Database at http://www.hiv.lanl.gov/ (accessed on 14 August 2009) after excluding 42 sequences with indels or due to their identity. The country representation of *gag* sequences included in the analysis is shown in Table 3, and included 431 sequences from South Africa, 49 sequences from Botswana, 41 sequences from India, 30 sequences from Zambia, 22 sequences from Israel, 18 sequences from Tanzania, 12 sequences from Ethiopia, 11 sequences from Malawi, and small numbers of sequences from Argentina (2), Brazil (6), China (2), Cyprus (5), Djibouti (1), Denmark (2), Spain (3), Georgia (1), Kenya (3), Senegal (1), Somalia (1), Uganda (2), USA (2), Uruguay (1), Yemen (1), and Zimbabwe (6). Sampling time of the retrieved sequences is also presented in Table 3. Most of the retrieved samples were sampled between 1998 and 2005. For analysis of viral dynamics the retrieved sequences were grouped by their sampling time. The 2007–2008 group included only five sequences, and was not used in the analysis of amino acid frequencies. The extended consensus sequence was built for each group. Frequencies of amino acid at each residue position were expressed as a fraction of 1. The low threshold of detection was 0.005.

*Viral diversity.* Retrieved HIV-1 subtype C *gag* nucleotide sequences were aligned using Muscle [54] followed by a BioEdit [55] manual adjustment. The maximum-likelihood (ML) method was used to estimate pairwise nucleotide distances. Evolutionary model was selected by using the Akaike information criterion in jModeltest 0.1.1 [56]. The parameters of the model (TPM1uf+I+Γ) were as follows: nucleotide frequencies, *f*_A_=0.4014, *f*_C_= 0.2286, *f*_G_=0.1962, and *f*_T_=0.1738; estimated value of shape parameter α of the Γ distribution = 0.5510; estimated value of proportion of invariable sites = 0.170; R matrix values, R_A↔C_ = 1.0; R_A↔G_ = 4.4966; R_A↔T_ = 0.6194; R_C↔G_ = 0.6194; R_C↔T_ = 4.4966; and R_G↔T_ = 1.0. The identified substitution model was used in PAUP* version 4.0b10 [57] to estimate ML-corrected pairwise distances. The majority consensus sequence for the earliest quasispecies was built in BioEdit.

*Phylogenetic analysis.* The genealogy reconstruction of the analyzed *gag* sequences was implemented in PhyML [23] using the HKY model of nucleotide substitution. The maximum likelihood tree was visualized by FigTree [58]. The potential disagreement between phylogenetic tree and phylogenetic network was tested by SplitsTree v4 [32,33] using the NeighborNet approach.

*Rates of non-synonymous and synonymous changes*. A subset of 138 *gag* sequences was selected by country representation (limit was set to three sequences per country per year of sampling). A single likelihood ancestor counting, SLAC, method was used as described in [38]. A global MG94 model was fitted for the entire *gag* alignment and was used for maximum likelihood reconstruction of ancestral codons. We inferred selection by SLAC using methods described in [59].

*Estimating time of the most recent common ancestor of HIV-1 subtype C.* The phylogeny and divergence time were estimated using the Bayesian MCMC inference under a ‘relaxed’ molecular clock model, as implemented in BEAST v1.4.8 [42,43]. Analysis was performed under an uncorrelated lognormal relaxed molecular clock model, using a general time-reversible nucleotide substitution model, estimated base frequencies, and heterogeneity among sites modeled with a gamma distribution. The demographic models of constant population size and Bayesian skyline plot were used. Alternative demographic models (exponential growth, expansion growth, and logistic growth) were not utilized because their use was shown to provide similar results in estimating time of the most recent common ancestor for HIV-1 group M [44]. Runs of 20 million steps each were performed, and the MCMC samples were inspected with Tracer v1.4.1 (Andrew Rambaut and Alexei Drummond), which indicated convergence and adequate mixing of the Markov chains, with high values of estimated sample sizes. The Bayesian skyline reconstruction was performed by Tracer v1.4.1.

*Statistical methods.* Data are summarized with medians (IQR). Comparisons between groups of sequences with different time of sampling were based on Mann-Whitney Rank Sum tests. Comparison of amino acid frequency in the subtype C extended consensus sequence was performed by the chi-square test for RxC tables and the Cochran-Armitage trend test. All reported p-values are 2-sided.

## 4. Conclusions

The study provides evidence for the overall stability of HIV-1 subtype C Gag among viruses circulating in the epidemic over the last decade. However, selected sites across HIV-1C Gag with changing amino acid frequency are likely to be under selection pressure on the population level. The time of the most recent common ancestor of HIV-1 subtype C viruses was dated to around 1950 (95% HPD 1928–1962).

## Figures and Tables

**Table 1.** Amino acid positions in HIV-1 subtype C Gag with frequency change ≥20% in the subtype C consensus sequence: potential association with known CTL epitopes. (Note: Epitopes with multiple changing amino acids are highlighted; changing amino acids are shown in bold and underscored).

Gag cleavage product	Amino acid position	Number of associated CTL epitopes	Epitope	HXB2		HIV-1 Subtype	HLA restriction
				start	end		
p17	**11**	**1**	ASILRG**G**KLDK	5	15	C	
	**28**	**10**	RLRPGGKK**H**Y	20	29	C	A*3002
			RLRPGGKK**H**YM	20	30	C	
			RPGGKK**H**Y	22	29	A, C, D	B42, B7
			RPGGKK**R**YM	22	30	C	B35, Cw*0602
			RPGGKK**K**YML	22	31	A, C, D	B*0702, B*5801, B*8101
			K**R**YMIKH**L**V	27	35	C	Cw*0602
			**H**YMLKH**I**VW	28	36	A, C	A*2301
	**34**	**7**	**H**YMLKH**L**VW	28	36	A, B, C	A*2301, A*2402, A24
			**H**YMLNH**I**VW	28	36	A, C, D	B*0702, B*5801, B*8101
			**H**YMLKH**L**VWAS	28	38	C	
			H**L**VWASREL	33	41	C	Cw*0602, Cw*0804
			**L**VWASRELERF	34	44	C	A*3002, A30, B*5703, B57
	**76**	**2**	EEL**R**SLYNTV	73	82	C	B*4006
			**R**SLYNTVATLY	76	86	B, C	A*30, A*3002, A30, B57, B58, B63
	**111**	**0**					
	**119**	**0**					
p24	**342**	**4**	RALGPGA**T**L	335	343	A, B, C, D	B7
			ALGPGA**S**LEEM	336	346	C	
			GPGA**T**LEEM	338	346	A, C, D	B*0702, B*5801, B*8101
			A**T**LEEMMTA	341	349	B, C, CRF01_AE	A*0201, A*0206, A*0220, A*0234, A*0236, A2
p2	**375**	**0**					

**Table 2.** Amino acid positions in HIV-1 subtype C Gag with frequency change 10% to 20% in the subtype C consensus sequence: potential association with known CTL epitopes (Note: Epitopes with multiple changing amino acids are highlighted; changing amino acids are shown in bold and underscored).

Gag cleavage product	Amino acid position	Number of associated CTL epitopes	Epitope	HXB2		HIV-1 Subtype	HLA restriction
				start	end		
p17	**7**	**1**	AS**I**LRGGKLD**K**	5	15	C	
	**15**	**2**	ELD**R**WE**K**IRL	12	21	B, C	B63
	**18**	**2**	**K**I**R**LRPGGKK	18	27	B, C, multiple	A*0301, A11, A3, B27
	**20**	**4**	**R**LRPGGKKHY	20	29	C	A*3002
	**30**	**7**	RLRPGGKKHY**M**	20	30	C	
			RPGGKKRY**M**	22	30	C	B35, Cw*0602
	**31**	**6**	RPGGKKKY**ML**	22	31	A, C, D	B*0702, B*5801, B*8101
			KRY**MI**KHLV	27	35	C	Cw*0602
			HY**ML**KHIVW	28	36	A, C	A*2301
			HY**ML**KHLVW	28	36	A, B, C	A*2301, A*2402, A24
			HY**ML**NHIVW	28	36	A, C, D	B*0702, B*5801, B*8101
			HY**ML**KHLVWAS	28	38	C	
	**61**	**0**					
	**62**	**0**					
	**65**	**0**					
	**69**	**0**					
	**79**	**6**	EELRSL**Y**NTV	73	82	C	B*4006
			RSL**Y**NTVATLY	76	86	B, C	A*30, A*3002, A30, B57, B58, B63
			SL**Y**NTVATL	77	85	A, B, C, CRF02_AG, D, F, G, K	A*02.01, A*0201, A*0202, A*0205, A*0214, A*0220, A*0234, A*0236, A*68, A02, A2, B*1503
			SL**F**NTVATLY	77	86	C	
			L**Y**NTVATLY	78	86	C	A*2902, A29, B*4403
			L**F**NTVATLY	78	86	C	A*2902
p17	**90**	**1**	YCVH**AG**I**E**V**R**D	86	96	C	
	**91**	**1**					
	**93**	**1**					
	**95**	**1**					
	**120**	**0**					
p24	**163**	**8**	VKVIEEK**A**F	156	164	C	B*1503
			VKVVEEK**A**F	156	164	B, C	B*1503
			IEEK**A**FSPEV	159	168	C	B*4006
			IEEK**A**FSPEVI	159	169	C	B*4501
			EEK**A**FSPEV	160	168	A, C	B*4415, B*4501
			EK**A**FSPEV	161	168	C	Cw*0602
			K**A**FSPEVIPMF	162	172	A, B, C, CRF02_AG, G	A*310102, A*6603, B*440302, B*5701, B*5703, B*5801, B57, B58, B63, B8, Cw*040101, Cw*07
			**A**FSPEVIPMFT	163	173	C	
	**215**	**3**	AAEWDR**L**HPVH	209	219	C	
			AEWDR**L**HPV	210	218	B, C	A2, B*04, B*4006, Cw*0602
	**223**	**4**	RLHPVHAGP**I**A	214	224	C	
			HPVHAGP**I**A	216	224	B, C	B*3910, B07, B35, B7
			HPVHAGP**V**A	216	224	A, B, C, D	B7
			GP**I**APGQM	221	228	C, D	B35
	**248**	**3**	TSTLQEQI**G**W	240	249	B, C, HIV-2	A*310102, A*6603, B*440302, B*5701, B*5703, B*58, B*5801, B27, B35, B57, B58, B63, B7, Cw*040101, Cw*07
			TSTLQEQI**A**W	240	249	B, C	B*5701, B*5703, B*5801, B57
			TLQEQI**G**WM	242	250	B, C	A*0201, A*0220, A*0234, A*0236, A2
	**260**	**6**	PPIPVG**D**IY	254	262	B, C	B*3501, B*3502, B35
			PPVPVG**D**IY	254	262	C	B35
			PPIPVG**E**IY	254	262	A, B, C, D	B35, B53, B7 supertype
			PVG**D**IYKRWII	257	267	C	
			G**E**IYKRWII	259	267	A, B, C, CRF02_AG, D	A*01, A*6801, B*0801, B*51, B8, Cw*07, Cw15, DQ2, DQ3, DR3, DR4
			**D**IYKRWII	260	267	B, C	B*0801, B8
	**286**	**0**					
	**312**	**3**	TLRAEQATQ**D**	303	312	C	Cw*0304
			RAEQATQ**D**VKN	305	315	C	
			QATQ**D**VKNW	308	316	C	B*5301, B*5801, B57
	**319**	**0**					
	**335**	**2**	NPDCKTIL**R**AL	327	337	C	B*3910
	**336**	**1**	**RA**LG**P**GATL	335	343	A, B, C, D	B7
	**339**	**3**	**A**LG**P**GASLEEM	336	346	C	
			G**P**GATLEEM	338	346	A, C, D	B*0702, B*5801, B*8101
p2	**370**	**1**	CLAEAMSQ**V**	362	370	B, C	A*0201, A*0220, A*0234, A*0236
	**371**	**0**					
	**374**	**0**					
p7	**377**	**0**					
	**380**	**0**					
	**381**	**0**					
	**389**	**0**					
	**401**	**1**	**I**AKNCRAPRKK	401	411	C	
p1	**441**	**1**	FLGKIWPS**H**K	433	442	A, B, C, CRF01_AE	A*0201, A*0205, A2
p6	**451**	**0**					
	**467**	**0**					
	**472**	**0**					
	**490**	**1**	PLTSL**K**SLFGS	485	495	C	
	**498**	**0**					

**Table 3.** HIV-1 subtype C *gag* sequences (>1,000 bp) included in the analyses by country of origin and by year of sampling. Countries with fewer than 10 sequences are presented as ‘Others’, and include Argentina (2), Brazil (6), China (2), Cyprus (5), Denmark (2), Djibouti (1), Georgia (1), Kenia (3), Senegal (1), Somalia (1), Spain (3), Uganda (2), USA (2), Uruguay (1), Yemen (1), and Zimbabwe (6). Groups of analyzed *gag* sequences by sampling year are outlined at the bottom.

**Country**	**n**	**1986**	**1987**	**1988**	**1989**	**1990**	**1991**	**1992**	**1993**	**1994**	**1995**	**1996**	**1997**	**1998**	**1999**	**2000**	**2001**	**2002**	**2003**	**2004**	**2005**	**2006**	**2007**	**2008**
**Botswana**	49											10		7	4	25							3	
**Ethiopia**	12	1		4								3	1				1	2						
**India**	41								4	5				2	23	4	2		1					
**Israel**	22													1	4	17								
**Malawi**	11								1							3	7							
**South Africa**	431												3	17	27	72	73	22	111	79	24	3		
**Tanzania**	18													2			11	5						
**Zambia**	30			1								1				6	11	8	3					
**Others:**	39				1	2	2	1						5	0	4	6	2	2	5	2	5	1	1
**Total:**	653	1	0	5	1	2	2	1	5	5	0	14	4	34	58	131	111	39	117	84	26	8	4	1
		**Before 2000:132 sequences**														**2000:131**	**2001–2002: 150**		**2003: 117**	**2004–2006: 118**				
